# Prune Consumption and Bone Health in Older Men: A One-Year Randomized Controlled Trial

**DOI:** 10.3390/nu18121854

**Published:** 2026-06-09

**Authors:** Lauren T. Ormsbee, Neda S. Akhavan, Joseph Munoz, Amy Mullins, Kelli S. George, Kallie E. Dawkins, Saiful Singar, Holly Clarke, Shalom Benton, Thomas Ledermann, Jeong-Su Kim, Michael Sweeney, Raedeh Basiri, Robert C. Hickner, Yinuo Zhang, Bahram H. Arjmandi

**Affiliations:** 1 Department of Health, Nutrition, and Food Sciences, College of Education, Health, and Human Sciences, Florida State University, Tallahassee, FL 32306, USA; lormsbee@fsu.edu (L.T.O.); rhickner@fsu.edu (R.C.H.); 2 Department of Kinesiology and Nutrition Sciences, School of Integrated Health Sciences, University of Nevada Las Vegas, Las Vegas, NV 89154, USA; 3 School of Agriculture and Food, Davis College of Agriculture and Natural Resources, West Virginia University, Morgantown, WV 26506, USA; 4 Center for Advancing Exercise and Nutrition Research on Aging (CAENRA), College of Human Sciences, Florida State University, Tallahassee, FL 32306, USA; 5 Department of Human Development and Family Science, College of Education, Health, and Human Sciences, Florida State University, Tallahassee, FL 32306, USA; 6 Department of Clinical Sciences, FSU College of Medicine, Florida State University, Tallahassee, FL 32306, USA; 7 Department of Nutrition and Food Studies, George Mason University, Fairfax, VA 22030, USA; 8 Institute of Sports Sciences and Medicine, College of Education, Health, and Human Sciences, Florida State University, Tallahassee, Fl 32304, USA

**Keywords:** prunes, osteopenia, DXA, supplementation, men’s bone health

## Abstract

**Background/Objectives**: Approximately 53.4 million U.S. adults aged 50 or older have low bone mass, yet male bone health remains under-researched. This study evaluated the effects of one year of prune supplementation on bone health in older men susceptible to, or with, osteopenia. **Methods**: A total of 59 men (aged 55–80 years) were randomly assigned to one of three groups: 100 g prunes, 50 g prunes, or 0 g prunes (control; multivitamin only) daily, with each group also receiving 450 mg elemental calcium and 800 IU vitamin D_3_ via a multivitamin. Dual-energy X-ray absorptiometry (Lunar model DXA; GE Healthcare, CA, USA) scans and blood samples were collected at baseline, 3 months, 6 months, and 12 months. **Results**: No significant changes were observed in total bone mineral density (BMD) or lumbar spine BMD over one year. There were no significant changes in C-reactive protein (CRP). Osteoprotegerin (OPG) decreased significantly in all groups; however, the decrease was significantly greater in the control group compared to the levels in both prune groups. Sclerostin (SOST) significantly increased over time within all groups. Tartrate-resistant acid phosphatase-5b (TRAP5b) increased in all groups, albeit in the control group, it increased significantly more over time compared to the increase in the 100 g group. **Conclusions**: Overall, prune supplementation, regardless of dosing, did not increase total or lumbar BMD or aid in maintaining bone density beyond the levels achieved by Ca++ and vitamin D_3_ supplementation in older men susceptible to, or with, osteopenia (with a negative T-score down to –2.5 standard deviations (SD) below the mean). Although between-group differences were observed in select secondary biomarkers (OPG, TRAP5b), these did not correspond to detectable changes in BMD and should therefore be considered exploratory rather than directly indicative of clinical bone benefit. Additional research is needed to fully understand the effects of prunes on bone metabolism in men.

## 1. Introduction

Osteoporosis is a debilitating degenerative disease characterized by decreases in bone mineral density (BMD) and quality, thereby increasing the risk of fractures, decreasing activities of daily living, and consequently raising the risk of morbidity and mortality [1,2,3,4]. In the United States, approximately 53.4 million individuals aged 50 or older suffer from low bone mass. Furthermore, 10 million of these individuals have diagnosed osteoporosis and of these, 2 million are men [1,3,5]. On average, men lose 0.5–1.0% of bone per year, beginning around the age of 60. Additionally, one in eight males over the age of 50 will incur an osteoporotic fracture, with men accounting for nearly 30 % of all hip fractures [6,7]. Moreover, men with osteoporosis have a higher mortality rate following a fracture, it is often underdiagnosed, and usually diagnosed later in men than in women [7,8]. Despite this knowledge, there is little research dedicated to male bone health.

As bone loss and osteoporosis progress, the bone matrix weakens and becomes brittle due to the process of bone remodeling and more specifically, due to a higher rate of bone resorption to bone turnover [9]. It is well documented that chronic inflammation and oxidative stress play a vital role in the development of this disease [3]. Inflammatory cytokines, including tumor necrosis factor (TNF) alpha, interleukin 1 (IL-1), and interleukin 6 (IL-6), can activate the osteoclastic activity that promotes bone resorption [10]. While there are targeted pharmacological treatment options (bone anabolics, anti-resorptives, etc.) to help combat the deleterious effects of this disease, they often come with unwanted side effects and/or poor adherence. These can include, but are not limited to, upper gastrointestinal distress, musculoskeletal pain, and skin and urinary infections [2,4]. Therefore, there is a need for alternative therapies to target the effects of chronic inflammation and subsequent bone loss to combat this disease.

There is ample evidence to support that certain fruits and vegetables contain various cardiovascular and bone-protective polyphenols that combat damaging free radicals, thus aiding in the reduction of chronic inflammation [11,12,13]. Specifically, prunes have been shown to have one of the highest oxygen radical absorbance capacities (ORAC), or antioxidant activity, in comparison to those for various other fruits and vegetables [11,13,14]. Previous research has demonstrated that prunes positively impact various physiological systems, including cardiovascular, neurological, and bone metabolism systems [15,16,17]. Regarding osteoporosis and bone health, there is evidence to support prunes’ bone-protective effects in animal models, as demonstrated by Muhlbauer et al. and others [14,18,19]. With respect to human studies, Hooshmand et al. and our current laboratory have produced positive findings for prune supplementation, including improved bone density in post-menopausal women [11,12,17]. Specifically, supplementation with 100 g of prunes, when compared to 75 g dried apples, daily demonstrated a significant improvement in BMD of the ulna and spine during a one-year intervention [12]. However, these same therapy interventions, particularly functional food supplementation with prunes, have not been fully assessed in a male population.

The current study aims to evaluate the effects of one year of prune supplementation on bone health in older men susceptible to, or with, osteopenia (a negative T-score up to −2.5 SD below the mean). Changes in total body and lumbar spine bone mineral density (BMD) were the primary outcome variables. Secondary outcome variables were biomarkers of inflammation and bone turnover.

## 2. Materials and Methods

### 2.1. Study Overview

This study is part of a series of investigations; therefore, the materials and methods have been similarly described by George et al. [3]. All individuals were recruited and screened from the greater Tallahassee area. For the present study, initial phone screenings were conducted to assess qualification and rule out any existing conditions/exclusion criteria. Following the phone screening, potential participants were scheduled for an on-site (Sandels Building Laboratory, College of Health and Human Sciences at Florida State University) screening that included both a written and verbal explanation of the informed consent, medical history, dietary and exercise questionnaires, and anthropometrics (height, weight, and waist [WC] and hip circumferences [HC]). Additionally, a DXA scan of the lumbar spine (L1–L4) was performed and analyzed by a licensed X-ray operator, under the supervision of a physician. A negative T-score BMD of −0.1 to −2.5 SD below the mean was needed for qualification, including those who were osteopenic but not osteoporotic. While T-scores of −0.1 to −0.9 are considered to be within the normal range, a negative T-score reflects bone mineral density below peak young-adult values and may indicate early reductions in relative bone strength, or weakening, prior to meeting diagnostic thresholds for osteopenia [20,21]. Therefore, we included any negative T-score up to −2.5 SD below the mean as a preventative intervention rather than including only those with clinical bone loss. According to the World Health Organization, the range of −1.0 to −2.5 SD is clinically indicative of osteopenia, whereas −2.5 SD below the mean or lower is clinically indicative of osteoporosis [22].

Upon enrollment, participants were assigned to one of three treatment groups: (1) 100 g prunes (high dose), (2) 50 g prunes (low dose), or (3) 0 g prunes (control [multivitamin only]; referred to as “control” in the remainder of the paper and figures). Each group was supplemented with a multivitamin containing 450 mg elemental Ca++ and 800 IU vitamin D_3_ to provide some bone protection during the one-year enrollment.

Participants reported to the FSU Sandels Laboratory between 6 am and 10 am for baseline, 3-month, 6-month, and 12-month visits, at which time the following data was collected: fasting venous blood draw, anthropometrics, 3-day dietary recall, and physical activity questionnaires. Additionally, DXA scans were performed for the total body and lumbar spine at the baseline, 3-month, 6-month, and 12-month visits. Upon completion of each visit, participants were provided with a 3-month supply of supplementation (dried prunes and a multivitamin containing calcium/vitamin D_3_), per their group assignment, an adverse events form to record any issues thought to be related to the intervention, and calendars to self-monitor compliance throughout the study. To avoid any gastrointestinal upset, particularly in the 100 g prune group, participants were advised to gradually increase daily consumption over the course of a week to allow the body to adjust. Participants were also given various recipe ideas to aid in compliance. All study procedures were approved by the Institutional Review Board (STUDY00000885) at the Florida State University (Tallahassee, FL, USA). This clinical trial was registered at ClinicalTrials.gov: https://clinicaltrials.gov/study/NCT03408119 (accessed on 27 May 2026).

### 2.2. Inclusion Criteria

Healthy men 55–80 years of age, with a lumbar (L1–L4) spine BMD T-score between −0.1 and −2.5 SD below the mean, were included. Men who were not taking pharmacological agents known to affect bone and who had not initiated an exercise program known to influence bone, particularly strength training, within the last 6 months were enrolled in the study. Men who met the inclusion criteria were considered for enrollment regardless of ethnicity or race.

### 2.3. Exclusion Criteria

Men who were receiving endocrine (e.g., prednisone, other glucocorticoids) or neuroactive (e.g., Dilantin, phenobarbital) drugs or any drugs known to influence bone and calcium metabolism were excluded from the study. Those who had initiated regular exercise regimens known to influence bone within the past 12 months prior to the study (e.g., resistance exercise training) were also excluded. Men whose BMD T-score at any site fell below −2.5 SD of the mean were excluded. Additionally, those treated with calcitonin, bisphosphonates, denosumab, raloxifene, anabolic agents (e.g., PTH and growth hormone), or steroids within a year prior to the start of the study were excluded. Men with metabolic bone disease, renal disease, cancer, cardiovascular disease, diabetes mellitus, respiratory disease, gastrointestinal disease, liver disease, or other chronic diseases were excluded. Potential participants with a body mass index (BMI) < 18 and >40 kg/m^2^ were excluded to avoid extremes in leanness/adiposity. Men who smoked 20 or more cigarettes per day and/or regularly consumed prunes or prune juice were also excluded.

### 2.4. Intervention

Qualified participants were randomly placed into one of three treatment groups: (A) 100 g prunes, (B) 50 g prunes, or (C) 0 g prunes (control; (multivitamin only)), determined by a computer-generated randomization sequence list. Previous research by Hooshmand et al. has shown doses of 50 g and 100 g to be well tolerated in the postmenopausal female population [10,11,16]. However, due to the laxative potential of prunes, participants were asked to gradually increase daily dosing at the start of the study. Each group was also supplemented with 450 mg elemental Ca^++^ (calcium diphosphate) and 800 IU vitamin D_3_ to provide some protection from bone loss throughout the duration of the study. Sample sizes per group were as follows: N = 23 in the 100 g group, N = 23 in the 50 g group, and N = 13 in the control multivitamin group.

The supplemental dosing of 450 mg elemental Ca^++^ (calcium diphosphate) and 800 IU vitamin D_3_ was determined based on current Recommended Dietary Allowances (RDA), (men aged 51–70, 1000 mg/day Ca^++^, 600 IU vitamin D_3_; men aged 71 and older, 1200 mg/day Ca^++^, 800 IU D_3_), as well as average daily intake from food for men of this age range [23]. Average calcium intake by American men typically exceeds 800 mg daily; therefore, with the additional 450 mg calcium via supplementation, participants likely met the RDA, though this was not directly measured, but should be in future studies. The average vitamin D_3_ intake from food is approximately 200 IU. Therefore, with the addition of 800 IU supplemental vitamin D_3_, participants likely met their RDA requirements during enrollment, though this was not directly measured, but should be in future studies [24].

Previous research supports that the current dosing of 50 g and 100 g of prunes demonstrates a positive influence on bone density, particularly in the ulna and lumbar spine, as well as on bone metabolism biomarkers, suggesting a suppression in the rate of bone turnover [12].

The prunes were donated and packaged in a snack-pack size by the California Prune Board. The Vitamin D_3_ and Ca^++^ were donated by Shaklee Corporation. All supplementation was provided free of cost to enrolled participants for the duration of the study.

### 2.5. Compliance

Compliance was monitored via a daily dosing calendar completed by the participants and returned at each time point. This allowed participants to mark missed doses and the reason for missing them. Participants were also contacted randomly throughout the study to encourage compliance and were asked to return any remaining prunes and unused calcium and vitamin D_3_ supplements at the 12-month final timepoint. Participants were required to meet >80% of their daily overall dosing to be considered compliant and to be included in the data set.

### 2.6. Questionnaires

#### 2.6.1. Health and Medical History

A detailed health and medical history was obtained during the screening and reviewed at the baseline visit to rule out any chronic conditions, medications, or bone health history that would be cause for exclusion from the study. If any new condition that qualified as an exclusion criterion presented during the duration of the enrollment, participants were removed from the study.

#### 2.6.2. Dietary Assessment

A 3-day food record was used to assess dietary intake. Clear instructions, along with a sample 3-day log and serving size guidelines, were given to each participant at the screening visit. Participants then completed the record prior to all testing days (baseline, 3-month, 6-month, and 12-month visits). Each record was reviewed and collected at subsequent visits. The record included two weekdays and one weekend day, to capture an estimate of average daily intake and consistency and to monitor compliance with the supplement. For analysis, the Food Processor, Version 11.11.32, database structure 11.11.10, was used (Salem, OR, USA).

#### 2.6.3. Physical Activity

The Five-City Project Physical Activity Questionnaire was used to assess physical activity patterns, as well as sleep patterns and duration, throughout the study timeline. At each time point, participants were asked to report total hours spent during the weekdays and weekend days on leisure, home activities (e.g., housework), and occupational tasks, as well as the intensity level of each activity, ranging from light to very intense [25]. Answers to questions regarding sleep hours during the weekdays and weekends were also recorded. The data was analyzed to determine consistency of activity and sleep throughout the enrollment. Initiation of an exercise training program, strength training in particular, during enrollment would have been grounds for exclusion and removal from the study.

### 2.7. Vitals

Resting blood pressure and resting heart rate (RHR) were recorded via an automated blood pressure/heart rate monitor (OMRON Hem 907XL IntelliSense Professional, Kyoto, Japan) following ten minutes of seated rest. Participants were instructed to sit upright with feet flat on the ground and left arm extended out at heart level with support. Three measurements were assessed, and the calculated mean recorded for statistical records.

### 2.8. Anthropometrics

Body weight was measured via a digital scale (Seca Corporation, Chino, CA, USA) at all time points to monitor any significant fluctuations in weight. Additionally, WC (measured via the horizonal plane midway between the lowest rib and the superior border of the right iliac crest) and HC (measured via the horizontal plane at the widest portion of the buttocks with the measuring tape parallel to the ground) were measured at each time point using a Gulick fiberglass measuring tape (Creative Health Products, Inc., Ann Arbor, MI, USA). For standardization purposes, measurements were taken with participants standing feet together and arms by their side. Measurements were taken twice to account for accuracy within 1 cm of each other, and the mean was recorded, along with the waist-to-hip ratio (W:H ratio).

### 2.9. Blood Collection and Processing

Fasting venous blood samples for plasma and serum analyses were collected via vacutainers at all time points. Samples were centrifuged at 3000× *g* for 15 min at 4 °C, aliquoted, and stored in a −80 °C freezer until analysis.

### 2.10. Bone Mineral Density (BMD)

BMD analyses were conducted by a certified X-ray technologist with physician oversight. Measurements for each subject included total BMD and lumbar spine using the Lunar iDXA (GE Healthcare, Madison, WI, USA). Scans were performed at baseline, 3-month, 6-month, and 12-month time points. All quality controls, scan positions, and analyses were accomplished according to the Lunar iDXA Operator’s Manual, with measurements performed using the “compare analysis” option.

### 2.11. Biomarkers of Inflammation

To assess the anti-inflammatory role of prunes, serum levels of CRP were assessed at all time points using commercially available ELISA kits (R & D Systems, Minneapolis, MN, USA).

### 2.12. Bone Biomarkers

Osteocalcin, SOST and TRAP-5b were assessed in duplicate at all time points (baseline, 3 months, 6 months, and 12 months) using commercially available ELISA kits (Quidel Biosystems, Mountain View, CA, USA; Bio-Techne, Minneapolis, MN, USA). SOST is a biomarker of osteocyte activity that acts to inhibit osteoblast activity.

TRAP5b is a biomarker of osteoclast activity, and increased levels have been correlated with increased bone resorption [26]. OPG, a biomarker of bone formation that acts as a RANK-L inhibitor [27], was assessed in duplicate at all time points using commercially available ELISA kits (Quidel Biosystems, Mountain View, CA, USA).

### 2.13. Statistical Analyses

The efficacy of the treatment was tested using Multilevel Growth Curve Modeling and the R package nlme [28]. This approach can handle missing data and allows residual variances to fluctuate across time points (i.e., not assuming homoscedasticity). Time was coded as 0, 1, 2, and 4 for baseline, 3-month, 6-month, and 12-month follow-up data, respectively. The control group was used as the reference group. For each outcome, we estimated a time effect, a group effect comparing the 100 g condition with the control condition, a group effect comparing the 50 g condition with the control condition, and two interaction effects testing the effect of time for each group. The restricted maximum likelihood (REML) estimation method was used to estimate the models. We estimated a series of models. First, we estimated a null model for each outcome to determine the intraclass correlation coefficient (ICC). We then estimated a growth curve model for each outcome. We used R (version 4.4.2) for descriptive statistics and the package ggplot2 [29] to visualize the results. Statistical significance was set at *p* < 0.05.

## 3. Results

### 3.1. Descriptives and Anthropometrics

**Figure 1:**
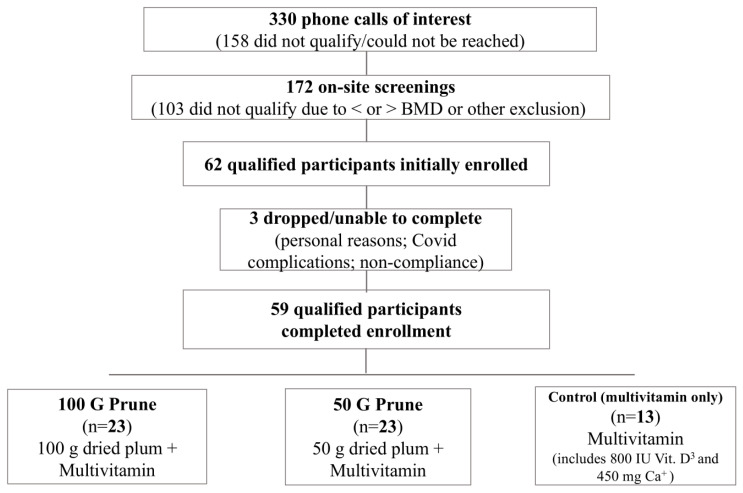
Screening/enrollment flowchart. (Bolded wording refers to the actual numbers and group naming while plain text wording provides explanation beneath.)

For the present study, 330 initial phone screenings were conducted to assess qualification and rule out any existing conditions/exclusion criteria; 172 potential participants qualified for an on-site screening. Of the 172 on-site screenings, 62 healthy men aged 55–80 years qualified and were enrolled (Figure 1). Three participants were unable to complete the final 12-month visit due to COVID-19 complications and on-campus research protocol restrictions. No adverse effects were reported beyond those anticipated and specified in the study protocol. A total of 59 men completed the full 12-month protocol (defined as completing the 12-month DXA scans). This sample size is sufficient to detect small-to-medium effect sizes (Cohen’s f = 0.17, alpha = 0.05, power = 0.8). The mean age of the participants was 67.0 y (SE = 0.9).

### 3.2. Anthropometrics and Vital Measurements

The means and SE for the anthropometric variables are shown in Table 1. The intraclass correlation coefficients across time were 0.99 for weight, 0.78 for body mass index (BMI), 0.91 for WC, 0.90 for HC, 0.02 for W:H ratio, 0.56 for RHR (in beats per minute), 0.60 for systolic blood pressure (SBP), and 0.62 for diastolic blood pressure (DBP).

Weight was significantly higher in the control group compared to in the 100 g group at baseline (b = −13.961, SE = 5.211, t(59) = −2.679, *p* = 0.01). BMI was significantly higher in the control group compared to the 100 g and 50 g groups at baseline (b = −5.280, SE = 1.552, t(58) = −3.402, *p* = 0.001; b = −4.356, SE = 1.589, t(58) = −2.742, *p* = 0.008, respectively). Waist measurements were significantly higher in the control group compared to in the 100 g group (b = −10.867, SE = 4.109, t (59) = −2.645, *p* = 0.011). RHR significantly decreased over time in the 50 g group (b = −2.589, SE = 1.162, t (164) = −2.228, *p* = 0.027). SBP significantly decreased over time in all groups (b = −4.286, SE = 1.351, t(164) = −3.172, *p* = 0.002), and in the 100 g group, it was significantly lower at all time points compared to in the 50 g and control groups ((b = −13.921, SE = 6.087, t(59) = −2.287, *p* = 0.026). It was also lower in both interaction terms (100 g: b = 3.892, SE = 1.617, t(164) = 2.406, *p* = 0.017; 50 g: b = 3.655, SE = 1.664, t(164) = 2.197, *p* = 0.029). No other significance was observed.

### 3.3. Dietary Intake

Table 2 provides the means and standard errors for the dietary intake variables. The intraclass correlations were 0.39 for calories, 0.26 for carbohydrates, 0.22 for fat, 0.33 for protein, 0.49 for cholesterol, 0.32 for sodium, 0.23 for sugar, 0.40 for fiber, 0.07 for saturated fat, 0.35 for polyunsaturated fat, 0.35 for monounsaturated fat, 0.09 for trans fat, and 0.16 for potassium. The MLM growth curve analysis, with time as a within-subject factor, revealed that there were no significant time effects for any dietary variables, i.e., b = 21.27, SE = 34.65, t(124) = 0.61, and *p* = 0.541 for calories; b = −1.08, SE = 5.06, t(124) = −0.21, and *p* = 0.831 for carbohydrates; b = −3.63, SE = 3.09, t(124) = −1.17, and *p* = 0.243 for fat; b = −0.68, SE = 1.72, t(124) = −0.40, and *p* = 0.692 for protein; b = 3.52, SE = 6.69, t(124) = 0.53, and *p* = 0.600 for cholesterol; b = −14.18, SE = 55.69, t(124) = −0.25, and *p* = 0.799 for sodium; b = −1.38, SE = 3.57, t(124) = −0.39, and *p* = 0.701 for sugar; b = −0.05, SE = 0.59, t(124) = −0.09, and *p* = 0.928 for fiber; b = 1.42, SE = 1.24, t(124) = 1.15, and *p* = 0.254 for saturated fat; b = 0.62, SE = 0.32, t(124) = 1.96, and *p* = 0.052 for polyunsaturated fat; b = 0.61, SE = 0.56, t(124) = 1.09, and *p* = 0.278 for monounsaturated fat; b = 0.03, SE = 0.04, t(124) = 0.61, and *p* = 0.542 for trans fat; and b = −44.79, SE = 92.66, t(117) = −0.48, *p* = 0.630 for potassium. All models included a random effect for time, except for the trans-fat model, which did not converge with a random time effect. 

### 3.4. Physical Activity

**Figure 2:**
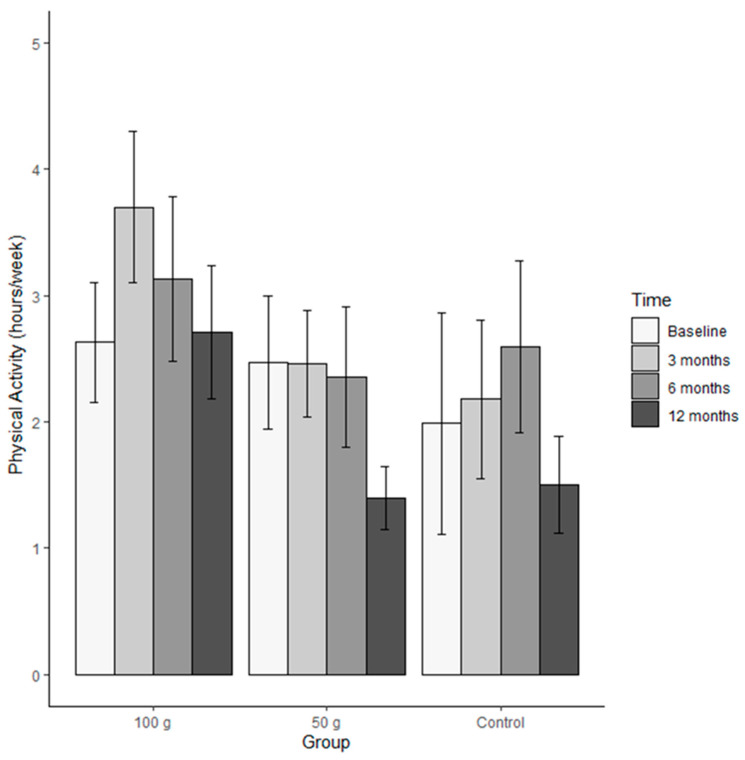
Physical activity per week in hours with standard errors.

The average hours of physical activity per week are shown in Figure 2. Bars represent mean physical activity (hrs/wk) (± SE) at baseline, 3-months, 6-months, and 12-months for each group (100 g prunes, 50 g prunes, and control). The ICC across time was 0.34. The MLM analysis revealed no significant effect for physical activity, b = 0.027, SE = 0.180, t(125) = 0.150, and *p* = 0.881 for time; b = 1.313, SE = 0.909, t(47) = 1.444, and *p* = 0.155 for group 100 g; b = 0.600, SE = 0.937, t(47) = 0.640, and *p* = 0.525 for group 50 g; b = −0.223, SE = 0.207, t(125) = −1.076, and *p* = 0.284 for the time by 100 g group interaction; and b = −0.340, SE = 0.212, t(125) = −1.605, and *p* = 0.111 for the time by 50 g group interaction. That is, the groups did not differ on average, and they did not change significantly.

### 3.5. Sleep Hours per Night

**Figure 3:**
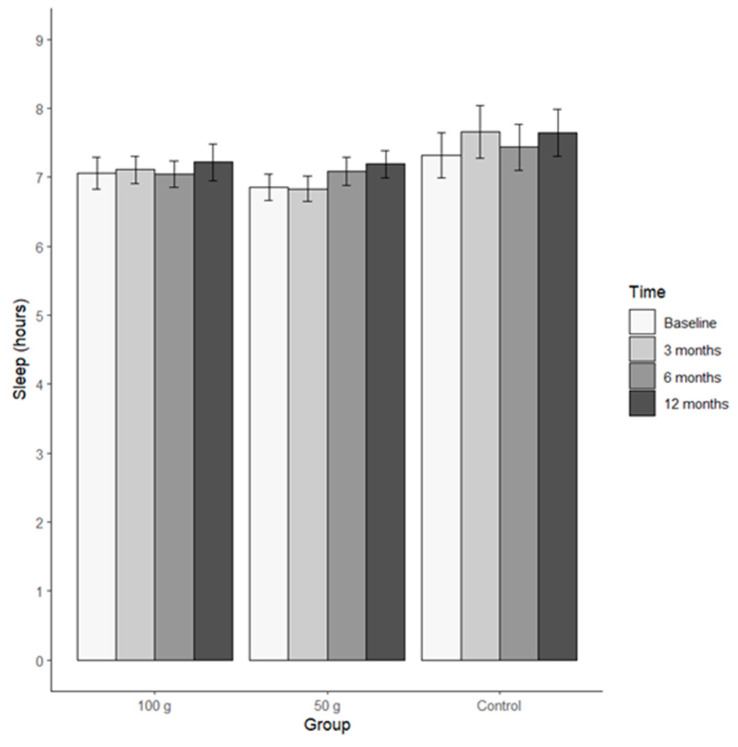
Average sleep per night in hours with standard errors.

Figure 3 illustrates the changes in average sleep per night. Bars represent mean hours of sleep per night (±SE) at baseline, 3 months, 6 months, and 12 months for each group (100 g prunes, 50 g prunes, and control). The ICC was 0.633. The MLM analysis revealed no significant effect for sleep, b = 0.111, SE = 0.074, t(133) = 1.500, and *p* = 0.136 for time; b = −0.278, SE = 0.347, t(47) = −0.802, and *p* = 0.427 for group 100 g; b = −0.502, SE = 0.356, t(47) = −1.411, and *p* = 0.165 for group 50 g; b = −0.085, SE = 0.086, t(133) = −0.998, and *p* = 0.320 for the time by 100 g group interaction; and b = −0.041, SE = 0.089, t(133) = −0.467, and *p* = 0.642 for the time by 50 g group interaction. These results indicate that the groups did not differ on average, and they did not change significantly.

### 3.6. Bone Mineral Density

**Figure 4:**
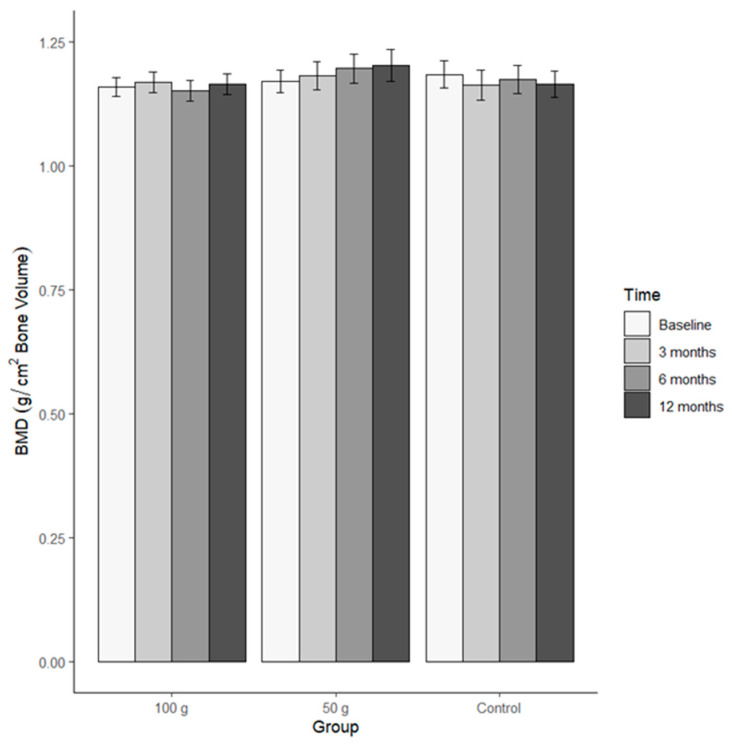
Average bone mineral density with standard errors.

The changes in bone mineral density are illustrated in Figure 4. Bars represent mean BMD (± SE) at baseline, 3 months, 6 months, and 12 months for each group (100 g prunes, 50 g prunes, and control). The ICC for bone mineral density was 0.929. The MLM analysis revealed no significant effect for BMD, indicating that the groups did not differ on average and did not change across time, b = −0.0003, SE = 0.003, t(168) = −0.103, and *p* = 0.918 for time; b = −0.022, SE = 0.035, t(67) = −0.630, and *p* = 0.530 for group 100 g; b = −0.013, SE = 0.035, t(67) = −0.366, and *p* = 0.716 for group 50 g; b = 0.004, SE = 0.004, t(168), = 1.061, and *p* = 0.290 for the time by 100 g group interaction; and b = 0.005, SE = 0.004, t(168), = 1.140, *p* = 0.256 for the time by 50 g group interaction.

### 3.7. DXA T-Score

**Figure 5:**
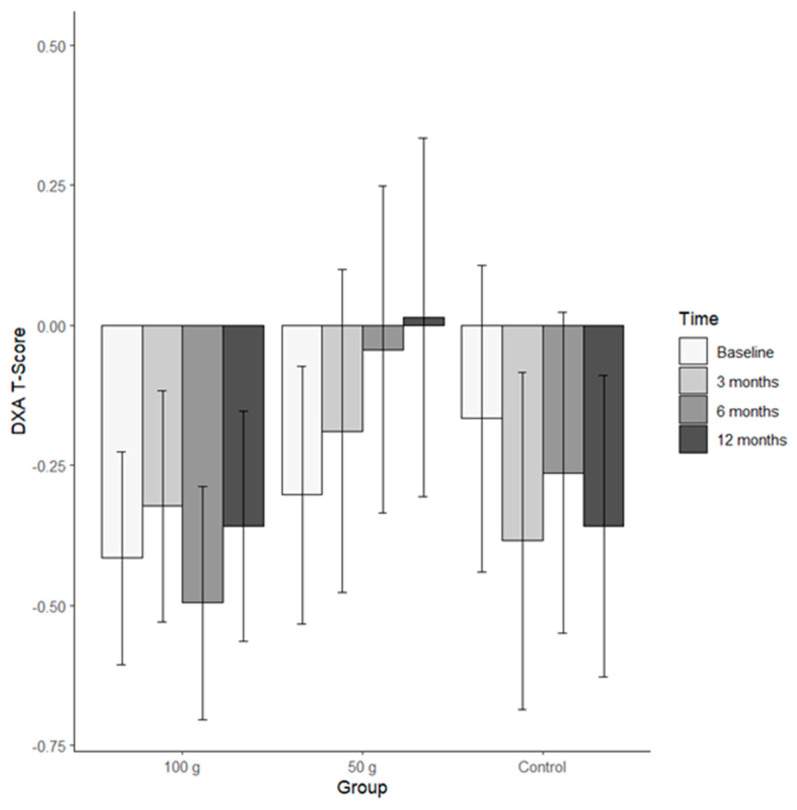
Average DXA T-scores with standard errors.

Figure 5 presents the changes in the DXA T-scores. Bars represent mean DXA T-scores (±SE) at baseline, 3 months, 6 months, and 12 months for each group (100 g prunes, 50 g prunes, and control). The ICC was the same as the BMD. As for BMD, the MLM analysis showed no significant effect on DXA T-scores, b = −0.003, SE = 0.031, t(168) = −0.101, and *p* = 0.920 for time; b = −0.219, SE = 0.346, t(67) = −0.635, and *p* = 0.528 for group 100 g; b = −0.126, SE = 0.346, t(67) = −0.365, and *p* = 0.716 for group 50 g; b = 0.040, SE = 0.038, t(168) = 1.050, and *p* = 0.295 for the time by 100 g group interaction; and b = 0.045, SE = 0.039, t(168) = 1.136, and *p* = 0.258 for the time by 50 g group interaction. That is, the groups did not differ on average, and there was no significant change. 

### 3.8. Lumbar BMD

**Figure 6:**
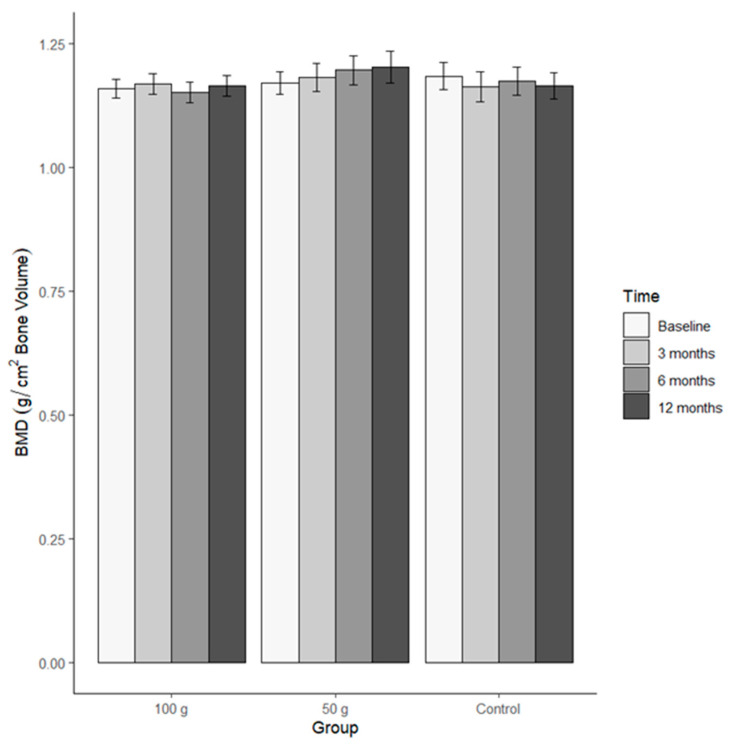
Average lumbar BMD with standard errors.

The changes in lumbar BMD are shown in Figure 6. Bars represent mean lumbar (± SE) at baseline, 3 months, and 6 months for each group (100 g prunes, 50 g prunes, and control). The ICC was 0.948. None of the effects were statistically significant, b = −0.003, SE = 0.031, t(168) = −0.101, and *p* = 0.920 for time; b = −0.219, SE = 0.346, t(67) = −0.635, and *p* = 0.528 for group 100 g; b = −0.126, SE = 0.346, t(67) = −0.365, and *p* = 0.716 for group 50 g; b = 0.040, SE = 0.038, t(168) = 1.050, and *p* = 0.295 for the time by 100 g group interaction; and b = 0.045, SE = 0.039, t(168) = 1.136, and *p* = 0.258 for the time by 50 g group interaction. That is, the groups did not differ on average, and there was no significant change.

### 3.9. Serum Blood Biomarkers

#### 3.9.1. High Sensitivity C-Reactive Protein

**Figure 7:**
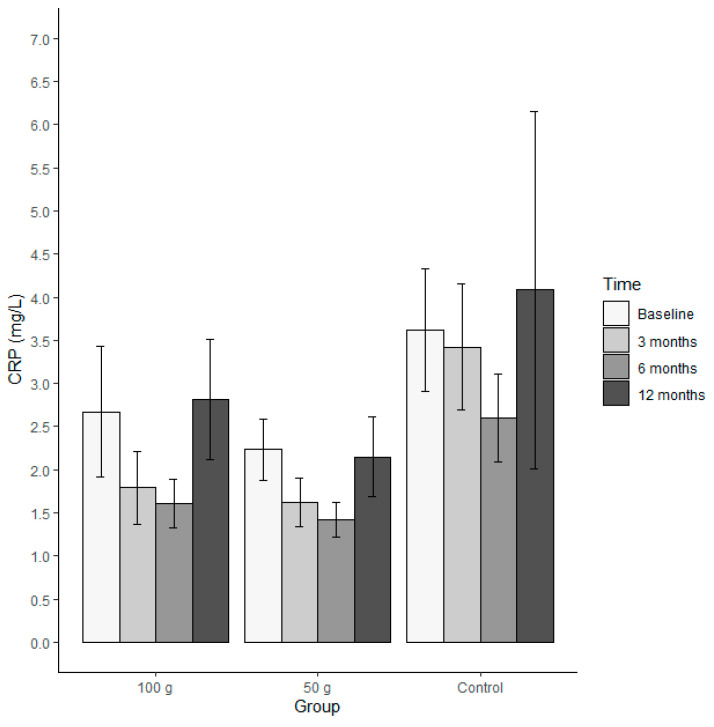
Average C-reactive protein with standard errors.

Figure 7 shows the average changes for CRP. Bars represent the mean CRP in mg/L (±SE) at baseline, 3 months, 6 months, and 12 months for each group (100 g prunes, 50 g prunes, and control). The ICC was 0.412. There was a significant effect for group 50 g, meaning that individuals in the control group had, on average, significantly higher levels of CRP than individuals in the group 50 g, b = −1.426, SE = 0.671, t (69) = −2.126, and *p* = 0.037. The other effects were not statistically significant, b = 0.257, SE = 0.210, t(175) = 1.220, and *p* = 0.224 for time; b = −1.172, SE = 0.669, t(69) = −1.751, and *p* = 0.084 for group 100 g; b = −0.242, SE = 0.252, t(175) = −0.963, and *p* = 0.337 for the time by 100 g group interaction; and b = −0.195, SE = 0.256, t(175) = −0.764, and *p* = 0.446 for the time by 50 g group interaction, suggesting that there was no significant change over time and no difference between the two treatment groups.

#### 3.9.2. Osteoprotegerin

**Figure 8:**
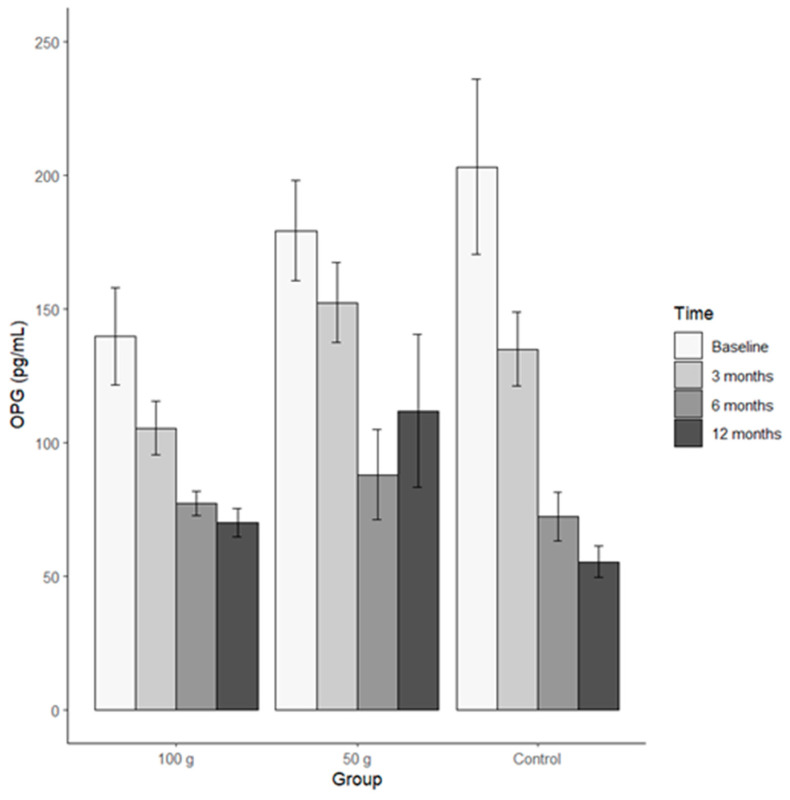
Average OPG with standard errors.

The average changes for OPG are shown in Figure 8. Bars represent mean OPG in pg/mL (± SE) at baseline, 3 months, 6 months, and 12 months for each group (100 g prunes, 50 g prunes, and control). For OPG, the ICC was 0.235. There was a significant time effect for OPG, and both time by group interaction effects were significant, b = −29.183, SE = 5.991, t(162) = −4.871, and *p* = 0.000 for time; b = 16.332, SE = 7.121, t(162) = 2.294, and *p* = 0.023 for the time by 100 g group interaction; and b = 21.124, SE = 7.352, t(162) = 2.873, and *p* = 0.005 for the time by 50 g group interaction. These results indicate that OPG significantly decreased and that this decrease was significantly stronger in the control group than in the 100 g and 50 g groups. There was also a significant group effect for the 100 g group, meaning that individuals in the 100 g group had, on average, significantly higher scores than individuals in the control group, b = −49.527, SE = 19.819, t (63) = −2.499, and *p* = 0.015. No such difference occurred between the 50 g group and the control group, b = −23.966, SE = 20.456, t (63) = −1.172, and *p* = 0.246.

#### 3.9.3. Osteocalcin

**Figure 9:**
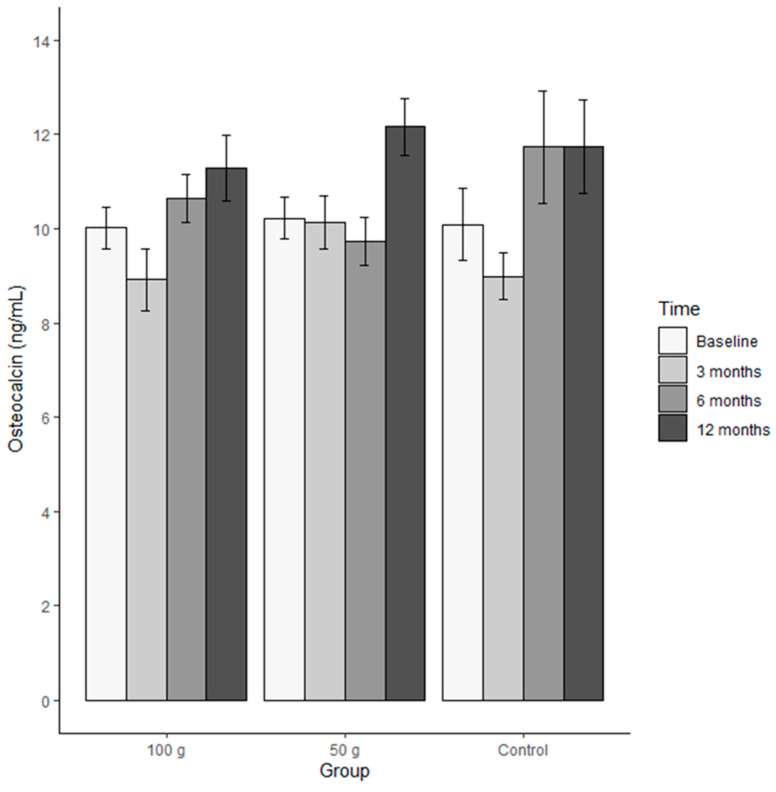
Average osteocalcin levels with standard errors.

The average osteocalcin levels are shown in Figure 9. Bars represent mean osteocalcin in ng/mL (±SE) at baseline, 3 months, 6 months, and 12 months for each group (100 g prunes, 50 g prunes, and control). The ICC for osteocalcin was 0.469. The MLM analysis revealed that none of the effects were statistically significant, b = 0.347, SE = 0.182, t(174) = 1.909, and *p* = 0.058 for time; b = −0.174, SE = 0.713, t(70) = −0.244, and *p* = 0.808 for group 100 g; b = 0.235, SE = 0.720, t(70) = 0.327, and *p* = 0.745 for group 50 g; b = 0.055, SE = 0.216, t(174) = 0.255, and *p* = 0.799 for the time by 100 g group interaction; and b = 0.126, SE = 0.221, t(174) = 0.570, and *p* = 0.570 for the time by 50 g group interaction. That is, there was no significant change and no significant group difference.

#### 3.9.4. Sclerostin

**Figure 10:**
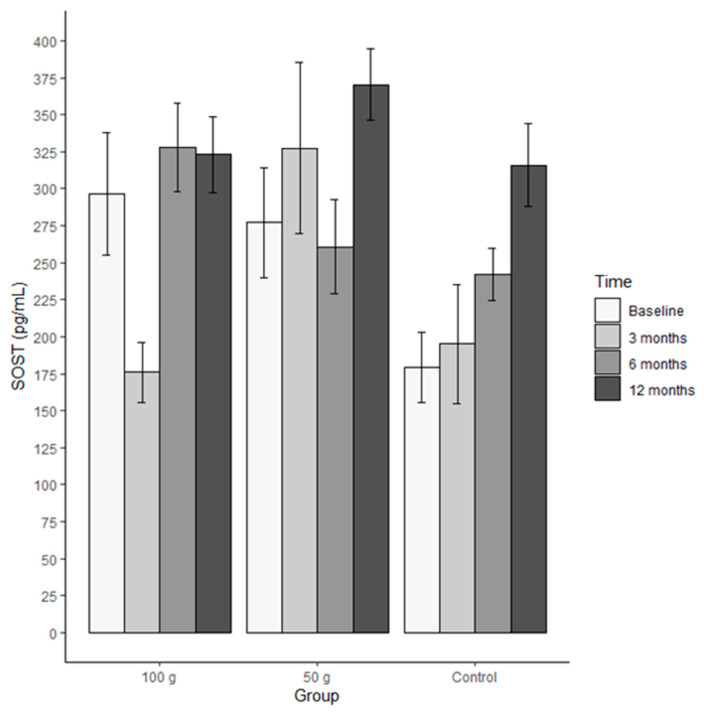
Average SOST with standard errors.

The average SOST levels are displayed in Figure 10. Bars represent mean SOST in pg/mL (±SE) at baseline, 3 months, 6 months, and 12 months for each group (100 g prunes, 50 g prunes, and control). There was a medium-sized ICC of 0.346 for SOST. There was a statistically significant time effect, indicating that there was a significant increase in SOST over time, b = 32.805, SE = 12.753, t (165) = 2.572, and *p* = 0.011. None of the other effects were significant, b = 75.325, SE = 58.375, t(63) = 1.290, and *p* = 0.202 for group 100 g; b = 85.323, SE = 59.926, t(63) = 1.424, and *p* = 0.159 for group 50 g; b = −19.358, SE = 15.225, t(165) = −1.271, and *p* = 0.205 for the time by 100 g group interaction; and b = −6.942, SE = 15.597, t(165) = −0.445, and *p* = 0.657 for the time by 50 g group interaction.

#### 3.9.5. Tartrate-Resistant Acid Phosphatase 5b

**Figure 11:**
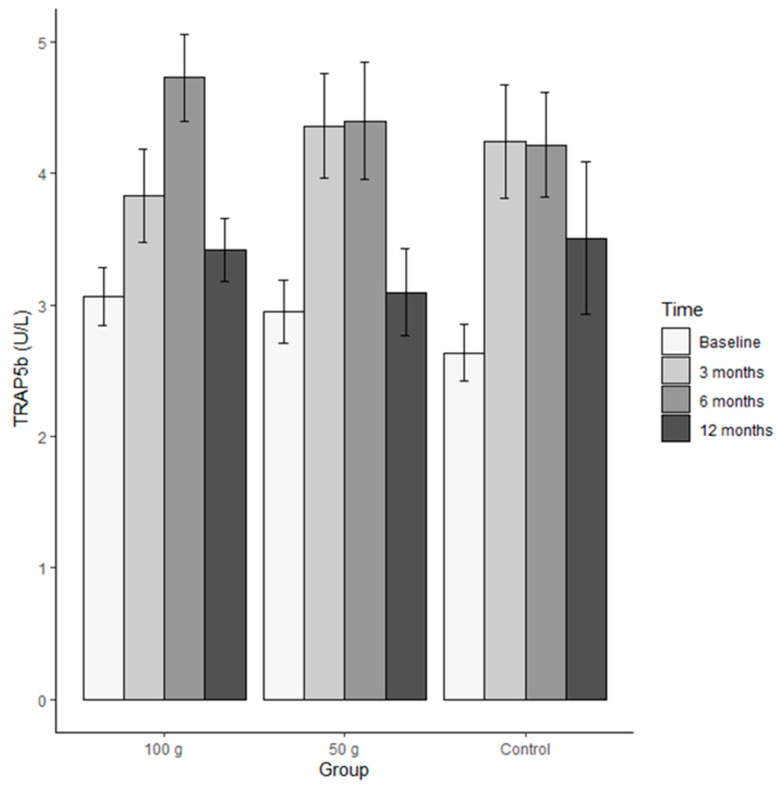
Average TRAP5b with standard errors.

The average change in TRAP5b is shown in Figure 11. Bars represent mean TRAP5b in U/L (±SE) at baseline, 3 months, 6 months, and 12 months for each group (100 g prunes, 50 g prunes, and control). The ICC for TRAP5b was 0.295. The MLM analysis revealed a significant time effect and a significant time by 100 g group interaction, b = 0.232, SE = 0.101, t (173) = 2.284, and *p* = 0.024; and b = −0.242, SE = 0.119, t (173) = −2.029, and *p* = 0.044, respectively. These results indicate an increase in all three groups, with a significant difference between the control and the 100 g group. The other effects were not significant, b = 0.537, SE = 0.398, t(69) = 1.350, and *p* = 0.182 for group 100 g; b = 0.415, SE = 0.406, t(69) = 1.023, and *p* = 0.310 for group 50 g; and b = −0.179, SE = 0.123, t(173) = −1.461, and *p* = 0.146 for the time by 50 g group interaction.

#### 3.9.6. Results Summary

There were no significant changes in total BMD or lumber BMD over the course of the 12-month study period. The changes were negligible, suggesting that the non-significant results were not due to insufficient statistical power [30]. CRP was significantly higher in the control group at all time points compared to the results for the 50 g prune group, while significance was not observed in any other group or over time. OPG significantly decreased over time in all groups; however, the decrease was significantly stronger in the control compared to in the 100 g and 50 g groups. In the 100 g group, OPG was, on average, significantly higher at all times compared to the levels for the control group. No significant change was observed for osteocalcin. SOST significantly increased over time in all groups. TRAP5b increased in all groups, although it increased significantly more over time in the control group compared to in the 100 g group.

## 4. Discussion

Though studies on prune consumption and bone health in men are scarce, our findings concur with the results of previous literature in that one year of supplementation does not yield improvements in total or lumbar spine BMD, nor aid in maintaining bone density beyond the levels achieved by elemental Ca^++^ and vitamin D_3_ supplementation in older men with a negative T-score between −0.1 and −2.5 SD below the mean. Although no significant increases in total BMD were observed, there was also no significant decline over the 12-month study period. This contrasts with the typical annual bone loss of 0.5–1.0% seen in men of this age group in real-world settings [6]. However, since the lack of measurable bone loss was observed in all groups, including the control, it cannot be directly attributed to prune supplementation. Interestingly, while data from Fajardo et al. also showed no significant increase in total BMD with 1-year prune supplementation, beneficial effects were observed with respect to changes in bone geometry detected by peripheral quantitative computed tomography (pQCT) [31]. This technique, used for BMD measurements in a peripheral part of the body, such as the forearm or leg, specifically showed increases in the periosteal and endosteal circumferences of the tibia [31]. In an animal model, Deyhim et al. noted bone-protective effects of dried plum via significant growth in trabecular bone microstructure and the preservation of tibial BMD [32]. Additionally, treatment with dried prunes at doses of 15% and 25% of the diet improved the BMD of the femur, as well as trabecular bone volume, which was also seen in an animal model [18]. These findings are encouraging in that, while total BMD may not be affected by prune supplementation over the course of one year, there may be a positive effect of prunes on bone strength and microstructure, thereby slowing bone loss in the long term. Additional research is needed to assess bone strength variables and their impact with respect to BMD in the current population.

While weight and BMI were significantly higher in the control group compared to in the 100 g and 50 groups, all participants were classified as “overweight” according to BMI classifications, and there was no significant difference between groups with respect to total or lumbar BMD. In the current study, a decrease in systolic blood pressure was observed over time in all groups, including in the control group, which did not receive prunes. This reduction may, in part, be due to the multivitamin that all groups received. Data has shown that supplementation with calcium and vitamin D_3_ can lower this variable, specifically in an overweight population [33,34]. The reduction in heart rate, seen only in the 50 g group, was a surprising finding and warrants further investigation into the effects of prunes on cardiovascular measures.

In the current study, the control (multivitamin only) group had significantly higher CRP concentrations, at all time points, compared to the levels in the 50 g prune group. The current literature is mixed regarding the effect of prune supplementation on CRP levels. While Hooshmand et al. [12] showed a significant reduction in CRP levels with one year of 100 g prune compared to apple supplementation in a post-menopausal female population, Damani et al. conducted a large-scale (N = 183) study and found no significant changes in CRP levels with one year of 50 g or 100 g of prune supplementation in a similar population. However, a decrease in proinflammatory cytokine secretion, particularly IL-6 and TNF-α, was observed, suggesting a decrease in inflammation [35]. A similar reduction in IL-6 and TNF-α was also observed by Hong et al. [36]. With particular respect to men, the few published studies concur with our findings in that 50 g and 100 g of prune supplementation does not significantly reduce levels of CRP [3,37].

OPG plays a vital role in bone health, aiding in the inhibition of osteoclastogenesis and bone resorption [27]. While the decrease over time in all groups in the current study is surprising, conflicting with findings from Hooshmand et al. [38], the decrease was significantly greater in the control group compared to in the 100 g and 50 g groups. Additionally, the 100 g group showed a significantly higher concentration of OPG across all time points in comparison to results for the control group. This difference was not observed between the 50 g and 100 g groups. However, these between-group differences in OPG did not correspond to any detectable differences in total or lumbar BMD. The clinical significance of the attenuated OPG decline in the prune groups therefore remains uncertain, and this finding should be regarded as exploratory. Whether the observed OPG pattern reflects a meaningful modulation of bone resorption pathways by prune-derived polyphenols or is the result of other bioactive compounds warrants further investigation.

Osteocalcin, secreted by osteoblasts, plays a direct and crucial role in directly binding calcium to bone and in aiding bone formation [39]. While a higher value in a younger population may indicate new bone formation, increases in an aging population can mean a high turnover rate, without increased bone strength, and high serum levels are correlated to rapid bone loss in osteoporotic patients [40]. The current study found no significant changes between groups or over time. Additional research is warranted to fully understand the possible effects of prunes on this variable.

SOST is an osteocyte-derived inhibitor that binds to the LRP5/6 receptor on the osteoblasts, thereby leading to a decrease in bone formation [9]. Thus, inhibiting sclerostin activity will not only promote bone formation but also inhibit bone resorption, resulting in maintenance of, and possibly improved, bone mineral density and bone homeostasis [41]. In osteopenia, where bone density is already compromised, increasing sclerostin levels would likely be detrimental, as this could further suppress bone formation and contribute to continued bone loss. Therefore, reducing sclerostin activity or inhibiting its expression may be beneficial in osteopenia to promote bone formation and improve bone density.

The current study results indicate that there was a significant increase in SOST across all groups over time. These results conflict with those in previous literature showing slight decreases in SOST following one year of prune supplementation, albeit these findings were in post-menopausal women [11,12]. Specifically, Hooshmand et al. observed a slight 1.1% decrease in SOST in a 100 g prune group compared to a 3.8% increase with dried apple supplementation, again in post-menopausal women [12]. That particular study, however, notes that a limitation of these findings is a small sample size, warranting further investigation. With respect to men, there is very little data available to compare with our results; however, George et al. reported no change in SOST levels over 3 months in this same male population, suggesting that the increase observed here may reflect a longer-term, age-related trajectory [3]. It is important to note that Mödder et al. demonstrated that serum sclerostin levels increase markedly with age in women but even more so in men, with lifespan values increasing 2.4- and 4.6-fold, respectively, and that the mechanism for metabolizing circulating sclerostin is undetermined [42]. Additionally, serum SOST levels are generally significantly higher in men compared to those in women and are significantly higher in older men compared to in their younger counterparts [42]. The results of the current study conflict with data for post-menopausal women, and with little data available for men, the findings warrant further research.

Higher levels of TRAP5b typically indicate increased bone resorption. In osteopenia, where there is already a risk of bone loss, an increase in TRAP5b could exacerbate the condition by promoting further breakdown of bone tissue. Previous literature by Fajardo et al. [31] demonstrated a time-dependent reduction in serum TRAP5b levels with one year of 100 g/day prune consumption (in a male population) at both the 3- and 6-month time point, whereas significance was not observed in the control group. Hooshmand et al. [38] demonstrated time-dependent reductions in TRAP5b, as well as in C-terminal collagen cross-link (CTX) levels, with 1-year prune supplementation at 3-month, 6-month, and 12-month intervals when compared with the baseline levels. While we would have expected to see similar findings in the current study, there was an increase over time in all groups, with a significantly lower increase over time in the 100 g group compared to in the control group. This attenuation is noteworthy; however, this biomarker difference did not translate into a detectable change in BMD between groups. As a secondary outcome, this finding should be interpreted as hypothesis-generating rather than as evidence of a clinically meaningful anti-resorptive effect. Whether the observed TRAP5b pattern would correspond to measurable bone preservation over a longer duration or in a larger, more homogeneous osteopenic population remains to be determined.

Limitations from this study include the COVID-19 pandemic, which caused a large reduction in recruitment number from that which was originally proposed. There were also large periods of time when the study was put on hold due to the World Health Organization (WHO) guidelines for age and high risk of COVID-19, thereby causing multiple missed time points and missing variables for certain participants, as well as dropped participants due to the severity and uncertainties of the virus. These limitations are also partly responsible for the imbalance of the final N per group, as the randomization order originally accounted for a sample size of 200, which had been sufficient to detect small effects (Cohen’s f = 0.09). In addition to a balanced design, it would also have been beneficial to have a true placebo group, who received no supplementation of any kind, to compare to the calcium/vitamin D3 and prune groups and to fully isolate any benefits from prune consumption. Additionally, individual variation in habitual dietary intake of these nutrients, which are critical determinants of BMD, may have introduced uncontrolled confounding results. Participants’ total calcium and vitamin D intake may have varied substantially, potentially masking or attenuating between-group differences in BMD. Additionally, this study recruited a heterogeneous population of men with a broader range of T-scores (−0.1 to −2.5 standard deviations below the mean) beyond that WHO-defined bone loss, therefore presents a limitation in assessing the effects of prunes on bone health in men. Future research should include a large-scale study specifically comprised of osteopenic men. While routine phone calls were made to participants to encourage supplement compliance, this study was self-reported, and as with intervention studies, there may be discrepancies between actual consumption vs. completed logging forms. Also, while we aimed to recruit a diverse population within Tallahassee and the surrounding areas, the enrolled participants do not fully represent the diversity within our community. Additionally, a larger-scale study of 100 or more individuals may shed more light on the effects of prunes on biomarkers of bone resorption and remodeling.

## 5. Conclusions

The results of the current study suggest that one year of prune supplementation did not increase total or lumbar spine BMD nor aid in maintaining bone density beyond the results of elemental Ca^++^ and vitamin D_3_ supplementation in older men susceptible to, or already diagnosed with, osteopenia. As with previous research in men, prunes, regardless of their dosing, did not decrease levels of CRP. TRAP5b increased in all groups, although it increased significantly more over time in the control group compared to the increase in the 100 g group. Additionally, there was a significant increase in SOST across all groups over time, with no significant differences in the rate of increase between the prune and control groups. Although between-group differences were observed in select secondary biomarkers (OPG, TRAP5b), these did not correspond to detectable changes in BMD and should therefore be considered exploratory rather than directly indicative of clinical bone benefit. Further larger-scale and longer-duration studies in this population are needed to fully assess the effect of prune supplementation on bone density and the biomarkers of inflammation and bone metabolism.

## Figures and Tables

**Table 1:** Means and standard errors for anthropometric and vital measurements from baseline to 12 months (N = 59).

Variable/Group	3-Month	6-Month	12-Month
Weight (kg)			
100 g Prunes	76.80 ± 2.27	76.07 ± 2.39	75.89 ± 2.52
50 g Prunes	81.87 ± 2.84	83.39 ± 3.09	81.05 ± 3.03
Control Group	90.83 ± 6.76	92.62 ± 8.19	90.62 ± 7.64
BMI (kg/m^2^)			
100 g Prunes	24.74 ± 0.72	24.30 ± 0.77	25.02 ± 0.71
50 g Prunes	26.34 ± 0.91	26.83 ± 0.99	25.44 ± 0.77
Control Group	29.22 ± 2.18	29.80 ± 2.63	27.01 ± 3.05
WC (cm)			
100 g Prunes	92.66 ± 2.55	93.34 ± 2.45	91.85 ± 2.43
50 g Prunes	96.50 ± 2.12	96.02 ± 2.59	94.81 ± 2.19
Control Group	104.79 ± 4.91	103.68 ± 5.54	103.81 ± 5.50
HC (cm)			
100 g Prunes	99.32 ± 1.30	98.35 ± 1.29	98.16 ± 1.61
50 g Prunes	103.74 ± 1.82	104.26 ± 1.91	102.30 ± 1.73
Control Group	107.26 ± 3.74	107.68 ± 4.33	107.55 ± 4.26
W:H Ratio			
100 g Prunes	0.941 ± 0.016	0.946 ± 0.016	0.935 ± 0.017
50 g Prunes	0.930 ± 0.011	0.920 ± 0.017	0.927 ± 0.015
Control Group	0.974 ± 0.020	0.961 ± 0.030	0.962 ± 0.030
RHR (bpm)			
100 g Prunes	62.69 ± 1.65	64.49 ± 1.75	63.55 ± 1.87
50 g Prunes	61.98 ± 2.49	59.11 ± 2.81	59.57 ± 1.85
Control Group	64.22 ± 2.81	66.46 ± 3.00	69.77 ± 7.03
SBP (mmHg)			
100 g Prunes	127.48 ± 3.77	129.70 ± 3.56	129.79 ± 4.29
50 g Prunes	133.14 ± 3.39	133.56 ± 3.12	133.52 ± 3.57
Control Group	138.42 ± 3.84	142.28 ± 7.93	124.55 ± 5.74
DBP (mmHg)			
100 g Prunes	75.28 ± 1.99	74.81 ± 2.00	73.29 ± 2.13
50 g Prunes	77.36 ± 2.26	76.11 ± 2.18	74.57 ± 2.06
Control Group	80.17 ± 3.79	82.02 ± 5.54	77.82 ± 4.94

**Table 2:** Dietary intake from baseline to 12 months (N = 59).

Variable	Baseline	3-Month	6-Month	12-Month
Calories (kcal)	2319.3 ± 126.9	2154.7 ± 122.8	2316.7 ± 180.2	2457.3 ± 108.2
Carbohydrates (g)	260.8 ± 19.0	264.5 ± 21.0	268.0 ± 32.6	279.7 ± 15.5
Fat (g)	114.7 ± 18.3	82.8.1 ± 6.1	99.4 ± 7.8	98.0 ± 4.6
Protein (g)	98.7 ± 5.9	96.1 ± 10.3	91.4 ± 6.3	101.0 ± 5.7
Cholesterol (mg)	322.8 ± 24.7	288.3 ± 37.8	341.3 ± 35.7	330.2 ± 25.4
Sodium (mg)	3885.2 ± 264.3	3220.4 ± 199.0	3686.7 ± 331.7	3841.4 ± 303.3
Sugar (g)	113.1 ± 16.0	106.6 ± 8.2	113.5 ± 18.7	123.1 ± 10.3
Fiber (g)	24.4 ± 1.6	24.7 ± 2.1	24.1 ± 3.0	25.9 ± 2.5
Saturated Fat (g)	27.5 ± 1.8	24.4 ± 2.3	29.9 ± 2.3	35.4 ± 5.7
Polyunsaturated Fat (g)	12.8 ± 0.97	14.2 ± 1.6	17.7 ± 2.0	15.0 ± 1.9
Monounsaturated Fat (g)	22.0 ± 2.1	22.2 ± 2.6	24.2 ± 1.8	24.2 ± 2.0
Trans Fat (g)	0.6 ± 0.2	0.7 ± 0.3	0.4 ± 0.1	0.4 ± 0.1
Potassium (mg)	3281.0 ± 382.1	2960.1 ± 243.8	3025.4 ± 386.1	2890.8 ± 252.9

## Data Availability

Data supporting these findings are available within the article or upon request.

## Abbreviations

The following abbreviations are used in this manuscript:

BMD	bone mineral density
CRP	C-reactive protein
SOST	sclerostin
OPG	osteoprotegerin
TRAP5b	tartrate-resistant acid phosphatase 5b
WC	waist circumference
HC	hip circumference
W:H ratio	waist-to-hip ratio
RHR	resting heart rate
BMI	body mass index
SBP	systolic blood pressure
DBP	diastolic blood pressure
IL-1	interleukin 1
IL-6	interleukin 6
